# Alterations of the Gut Microbiota Associated With Promoting Efficacy of Prednisone by Bromofuranone in MRL/lpr Mice

**DOI:** 10.3389/fmicb.2019.00978

**Published:** 2019-05-01

**Authors:** Zhixing He, Xiangyu Kong, Tiejuan Shao, Yun Zhang, Chengping Wen

**Affiliations:** Institute of Basic Research in Clinical Medicine, College of Basic Medical Science, Zhejiang Chinese Medical University, Hangzhou, China

**Keywords:** gut microbiota, MRL/lpr mice, prednisone, bromofuranone, AI-2/LuxS quorum sensing

## Abstract

Gut microbiota played an important role in systemic lupus erythematosus (SLE) and glucocorticoids were prone to cause alterations in gut microbiota. This study addressed the effect of bromofuranone on the treatment of SLE with prednisone, since bromofuranone could regulate gut microbiota by inhibiting the AI-2/LuxS quorum-sensing. Remarkably, bromofuranone did not alleviate lupus but promoted the efficacy of prednisone in the treatment of lupus. The alterations in the gut microbiota, including decreased *Mucispirillum*, *Oscillospira*, *Bilophila* and *Rikenella*, and increased *Anaerostipes*, were associated with prednisone treatment for SLE. In addition, the increase of *Lactobacillus*, *Allobaculum*, *Sutterella*, and *Adlercreutzia* was positively associated with the bromofuranone-mediated promotion for the treatment of lupus. This was the first study demonstrating that the efficacy of glucocorticoids could be affected by the interventions in gut microbiota.

## Introduction

Systemic lupus erythematosus was a severe multisystemic autoimmune disease characterized by the loss of tolerance to autoantigens along with the production of antinuclear antibodies. Autoantibodies produced by autoreactive B cells leaded to the formation and deposition of immune complexes in most tissues of the body (Sanz and Lee, 2010). The etiology of SLE had been related to genetic, environmental, hormonal, and immunological factors (Tsokos, 2011). Recently, a strong relationship between the gut microbiota and SLE had been demonstrated in SLE patients (Hevia et al., 2014; He et al., 2016) and lupus mice (Zhang et al., 2014; Luo X.M. et al., 2018). Furthermore, there was evidence that the gut microbiota played an important role in the regulation of anti-nuclear antibodies, TLR2/IL-17, IFN-γ, TLR4, Th17/IgM, etc. (Mu et al., 2015). Therefore, the gut microbiota could affect the progression of SLE.

Glucocorticoids were the most effective anti-inflammatory immunosuppressant and the cornerstone for the treatment of SLE. Physiological and pharmacological levels of GCs could protect intestinal mucosa from chemical substances, enzyme, and microbial damage through promoting the synthesis and secretion of mucins (Das et al., 2013; Li et al., 2016). Increasing evidences had shown that some signaling pathways related to GCs were due to their effects on gut microbiota (Buren et al., 2008; Wu et al., 2018). Hence, GCs might regulate physiological effects through gut microbiota. In addition, gut microbiota could transform GCs (Ridlon et al., 2013; Morris and Ridlon, 2017) and regulate host’s behaviors through glucocorticoid receptor pathway genes (Luo Y.Y. et al., 2018). Thus, gut microbiota might effectively influence SLE treatment with GCs.

Quorum sensing, the ability to communicate and regulate group behavior among the members of the gut microbiota (Waters and Bassler, 2005), could shape the composition of gut microbiota. Increasing evidence revealed that QS dominated critical physiological processes in the intestine and affected the virulence processes of invading pathogens (Thompson et al., 2015; Kamareddine et al., 2018). Multiple quorum-sensing signals were species-specific; however, autoinducer-2 (AI-2) signal was common throughout the bacterial kingdom (Pereira et al., 2013). It had been proved that AI-2 could affect invasive pathogens (Hsiao et al., 2014) and regulate the abundance of the major species in the intestines (Thompson et al., 2015). Overall, inhibiting AI-2 signals was an effective way to regulate gut microbiota compositions in the intestine.

Bromofuranone, a well-known inhibitor of AI-2 quorum sensing (Sivakumar et al., 2019), was used to regulate gut microbiota in MRL/lpr mice. The purpose of this study was to demonstrate the effects of bromofuranone on the efficacy of GCs for SLE.

## Materials and Methods

### Mice

MRL/lpr mice (8 weeks old) were purchased from Shanghai SLAC Laboratory Animal Co., Ltd., and maintained in the specific-pathogen-free environment of Zhejiang Chinese Medical University laboratory animal research center. After 7 days of adaptive feeding, the MRL/lpr mice were randomly divided into four groups (five per group): PT (oral gavage with 5 mg prednisone/kg of body per day), BT (oral gavage with 2 mg bromofuranone/kg of body per day), PBT (oral gavage with 5 mg prednisone and 2 mg bromofuranone/kg of body per day), and MT (oral gavage with sterile water per day). All mice were housed under a 12 h/12 h light/dark cycle and constant temperature (25 ± 1°C) and humidity (50 ± 5%) with food and water available *ad libitum*. Both prednisone (purity ≥ 99.0%, Sigma-Aldrich, United States) and bromofuranone (purity ≥ 97.0%, Sigma-Aldrich, United States) were administered from 9 to 14 weeks of age. Mice were weighed twice weekly, and the drug doses were adjusted accordingly. All animal experiments were performed according to the requirements of the Institutional Animal Care and Use Committee of China.

After the 5-week treatment, samples were collected at 12 h after the last drug administration. Blood was obtained from the eye socket vein in each mouse and centrifuged at 1300 *g* for 10 min at 4°C for serum. The fecal material was removed from the colon and stored at -80°C for further analysis and kidneys were stored in paraformaldehyde solution or directly stored at -80°C.

### Evaluation of Lupus Activity in MRL/lpr Mice

In this study, the lupus activity is determined by serum indexes and kidney pathology defined by hematoxylin-eosin (HE) staining. Serum Cr and BUN were measured based on an enzymatic-colorimetric method by using standard test kits on TBA-40FR automatic biochemical analyzer (Toshiba Medical Sys-terms Co., Ltd., Tokyo, Japan). The serum titer of anti-dsDNA autoantibodies and IFN-α were respectively measured by using mouse anti-dsDNA ELISA Kit (Shibayagi Co., Ltd, Japan) and mouse IFN alpha ELISA Kit (Invitrogen by Thermo Fisher Scientific) according to the manufacturers’ instructions.

### Detection of AI-2 Activity

AI-2 signals in the MRL/lpr mice stool were extracted as previously described (Thompson et al., 2015). Stool samples were homogenized at the concentration of 10% (weight/volume) in 0.1 M MOPS (C_7_H_15_NO_4_S 14.95 g/L, NaOH 1.14 g/L). The homogenized samples were centrifuged and filtered, and then an equal volume of methanol was added to precipitate remaining debris. The supernatants were vacuum-dried, resuspended in sterile water at the concentration of (50% weight/volume), and analyzed by bioassay.

AI-2 activity was measured using the *Vibrio harveyi* AI-2 reporter strain B170 as previously described (Bassler et al., 1997). The reporter strain B170 diluted at a ratio of 1:5000 with fresh medium and cultured for 12 h at 30°C in AB medium, and 100 μl of the diluted cells were then added to microtiter wells containing 100 μl of different substances before the test of AI-2 activity. The microtiter plates were shaken at 140 rpm at 30°C in a rotary shaker for 12 h followed by light production measured by fluorescence microplate reader. The induction of luminescence by each tested supernatant was determined by the relative change to that of the negative control of sterile AB medium instead of culture supernatant.

### DNA Extraction, 16S rRNA Gene Amplification and Sequencing

Total DNA was extracted from stool samples of 20 mice using the QIAamp^®^DNA Stool Mini Kit (Qiagen, Hilden, Germany) according to the manufacturer’s protocols. DNA extracts were determined by agarose gel electrophoresis (1% w/v agarose) and quantified using a NanoDrop 2000 spectrophotometer (Thermo Fisher Scientific).

The V3-V4 region of the 16S rRNA gene was amplified by PCR using a 30 μl mixture containing 0.5 μl of DMSO, 1.0 μl of 319F (10 mM), 1.0 μl of 806R (10 mM), 5.0 μl of the DNA sample, 7.5 μl of ddH_2_O, and 15.0 μl of Phusion High-Fidelity PCR Master Mix with HF Buffer (NEB) (He et al., 2016). The reactions were hot-started at 98°C for 30 s, followed by 30 cycles of 98°C for 15 s, 58°C for 15 s, and 72°C for 15 s, with a final extension step at 72°C for 1 min. Subsequently, the amplicons were purified according to standard procedures, quantified, pooled and sequenced with the MiSeq Reagent Kits v3 (600 cycles, Illumina) according to the manufacturer’s instructions with 20% OhiX (Illumina). The sequencing reaction was conducted by Hangzhou Guhe Information and Technology Co., Ltd., Zhejiang, China.

### Data Analysis and Bioinformatics

After sequencing, generated FASTQ data of 20 mice were prepared for analysis using Quantitative Insights Into Microbial Ecology (QIIME, version 1.9) (Caporaso et al., 2010). Clean reads were extracted from the raw paired-end reads according to the following criteria: (i) reads were truncated at any site receiving an average quality score of <20 bp over a 50-bp sliding window, and truncated reads shorter than 50 bp were discarded; (ii) exact barcode matching, two nucleotide mismatch in primer matching, and reads containing ambiguous characters were removed; (iii) only sequences that overlapped for more than 20 bp were merged according to their overlap sequence, reads that could not be merged were discarded.

Clean reads were clustered into the 16S rRNA Operational Taxonomic Units (OTUs) with a 97% similarity cutoff using UCLUST (Edgar, 2010). Taxonomic assignment was performed using the SILVA database (Quast et al., 2013), and the necessary sequences were blasted in the NCBI database for further classification (Sayers et al., 2018). The OTUs with less than 0.005% sequences of the total number of reads, or present in one sample, were filtered out. The statistical comparisons of alpha-diversity metrics were performed using the R programme package “Vegan.” The calculated beta-diversity metrics (unweighted UniFrac) were compared using the nonparametric ANOSIM measure. Principal coordinate analyses based on the beta-diversity metrics were conducted using the R package. The specific characterization of the gut microbiota was analyzed using the linear discriminant analysis (LDA) effect size (LEfSe) method^1^ (Segata et al., 2011). LEfSe by the Kruskal–Wallis test was carried out to determine the features of significantly different abundances among the treated groups and the effect size of each feature was accessed by LDA. All features identified by LEfSe exceeded an LDA score of 2.0, which indicates a significant difference between the two groups. *T*-test (soft GraphPad Prism 5) was conducted to verify the significantly different genera (LDA > 2.0 and relative abundance > 0.1%) between two groups based on the LEfSe results. Additionally, Spearman’s rank correlation was conducted between the indexes of lupus activity and altered microbial genera that were found to be significantly different between groups in 20 mice.

Differences in diversity indexes and serum indexes among multiple groups were analyzed using the Mann-Whitney nonparametric test by software SPSS 16.0 in 20 mice. Following statistical analyses with multiple comparisons, *p*-Values were adjusted using the Benjamini-Hochberg method to correct the false discovery rate (FDR).

## Results

### Lupus Activity in MRL/lpr Mice

**FIGURE 1:**
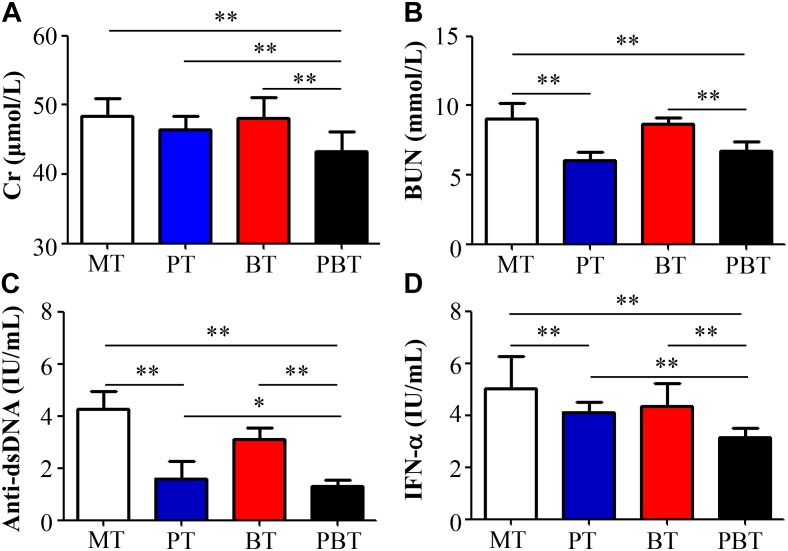
Serum creatinine **(A)**, uric acid nitrogen **(B)**, anti-dsDNA **(C)**, and INF-α **(D)** levels of MRL/lpr mice of four groups. MT, the model group; PT, the prednisone-treated group; BT, the bromofuranone-treated group; PBT, the combined drugs-treated group. “^∗^”: represents the adjusted *p*-Value < 0.05 between two groups; “^∗∗^”: represents the adjusted *p*-Value < 0.01 between two groups.

Like SLE patients, lupus activity of MRL/lpr mice could be associated with the four indexes shown in Figure 1. Compared with the model MRL/lpr mice, serum titer of BUN, anti-dsDNA and IFN-α were significantly reduced in prednisone-treated and combined drugs-treated mice (Figure 1B–D). Serum Cr concentration was significantly reduced by combination use of prednisone and bromofuranone but not by prednisone alone (Figure 1A). However, no significant difference was observed between MT and BT groups. Renal pathology indicated that mice of MT group developed the most severe lupus activity among the four groups (Supplementary Figure S1).

To further reveal the effect of combination therapy, this study also made a comparison among PT, BT, and PBT groups. Notably, compared with prednisone-treated and bromofuranone-treated mice, serum titer of Cr, anti-dsDNA autoantibodies and INF-α were significantly reduced in the combination-treated mice (Figure 1A,C,D).

In summary, bromofuranone had no effect on relieving lupus activity but could promote the treatment efficacy of prednisone.

### Diversity of Gut Microbiota in MRL/lpr Mice

**FIGURE 2:**
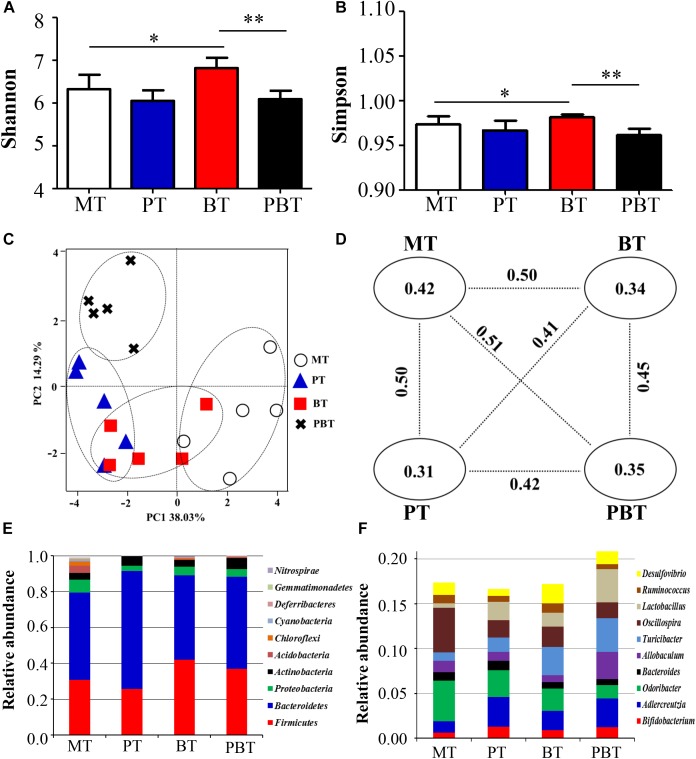
Alpha diversity indexes [Shannon **(A)**; Simpson **(B)**], beta diversity [PCoA score plots **(C)**; unweighted UniFrac distance **(D)**] and identified major phylum **(E)** and genus **(F)** in MRL/lpr mice of four groups. MT, the model group; PT, the prednisone-treated group; BT, the bromofuranone-treated group; PBT, the combined drugs-treated group. “^∗^”: represents the adjusted *p*-Value < 0.05 between two groups; “^∗∗^”: represents the adjusted *p*-Value < 0.01 between two groups.

To assess the overall difference of microbial compositions among the four groups, measurement of alpha- and beta- diversity was calculated. As shown in Figure 2A,B, Shannon and Simpson, the common alpha-diversity measurements of richness and evenness, were compared among groups. Only bromofuranone caused a significant increase in Shannon and Simpson in MRL/lpr mice, but neither prednisone nor the combined drugs had a noteworthy effect. In addition, there was a significant difference in alpha-diversity of the gut microbiota between bromofuranone-treated mice and combined drugs-treated mice (Figure 2A,B).

The beta-diversity was evaluated using unweighted UniFrac metric in the four groups. A scatter plot based on PCoA scores showed a clear separation of the community composition among the four groups (Figure 2C). PC1 and PC2 explained 38.03 and 14.29% of the total variance of microbial species, respectively. Figure 2D shows the unweighted UniFrac distance values between the two groups. The greatest intergroup distance value was observed between MT and PBT groups (value = 0.51) (Figure 2D) and all intragroup distance values were lower than the intergroup distance values, indicating good repeatability.

At the phylum level, *Bacteroidetes* appeared to be the most abundant in all four groups, followed by *Firmicutes*, *Proteobacteria*, and *Actinobacteria* (Figure 2E). MT had the lowest percentage of *Bacteroidetes* and *Firmicutes* (79.5%) and PT had the most significant proportion of *Bacteroidetes* and *Firmicutes* (91.5%). At the genus level, the top ten genera in abundance were *Bifidobacterium*, *Adlercreutzia*, *Odoribacter*, *Bacteroides*, *Allobaculum*, *Turicibacter*, *Oscillospira*, *Lactobacillus*, *Ruminococcus*, and *Desulfovibrio*. The total percentage of top ten genera were 17.4, 16.7, 17.2, and 20.9% in MT, PT, BT, and PBT groups, respectively (Figure 2F).

Compared with MT, gut microbiota in the three groups all changed, but with the inconsistent tendency. Notably, bromofuranone significantly increased the diversity of gut microbiota.

### Alterations in Microbial Genera Associated With Prednisone

**FIGURE 3:**
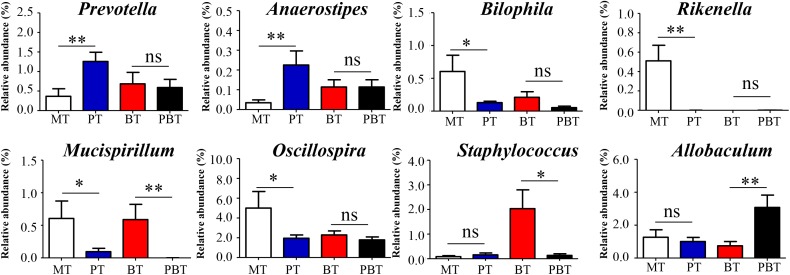
Significantly altered genera are associated with prednisone. MT, the model group; PT, the prednisone-treated group; BT, the bromofuranone-treated group; PBT, the combined drugs-treated group. “^∗^”: represents the adjusted *p*-Value < 0.05 between two groups; “^∗∗^”: represents the adjusted *p*-Value < 0.01 between two groups; “ns”: represents the adjusted *p*-Value > 0.05 between two groups.

To investigate the prednisone-associated alterations of gut microbiota, two comparisons (MT vs. PT and BT vs. PBT) were performed using the LEfSe analysis. Compared with MT group, 33 bacterial taxa in total were significantly altered in the PT group (Supplementary Figure S2A). At the phylum level, prednisone caused a significant decrease in Proteobacteria and Deferribacteres (Supplementary Figure S2A). To clearly illustrate the main alterations associated with prednisone, Figure 3 showed the significantly altered genera (*p* < 0.05, *t*-test) with relative abundance > 0.1%. Prednisone caused the significant increase in *Prevotella* (from 0.40 to 1.30%, *p* = 0.009) and *Anaerostipes* (from 0.04 to 0.20%, *p* = 0.015) and decrease in *Rikenella* (from 0.51 to 0.0%, *p* < 0.001), *Mucispirillum* (from 0.60 to 0.10%, *p* = 0.045), *Oscillospira* (from 5.00 to 1.90%, *p* = 0.039) and *Bilophila* (from 0.60 to 0.13%, *p* = 0.043) (Figure 3).

Prednisone was the only variable in comparison of the BT and PBT groups. Compared with the BT group, 21 bacterial taxa were significantly altered in the PBT group (Supplementary Figure S2B). The significantly altered genera between the BT and PBT groups were *Allobaculum* (0.70% in BT and 3.10% in PBT, *p* = 0.009), *Staphylococcus* (2.00% in BT and 0.13% in PBT, *p* = 0.018) and *Mucispirillum* (0.59% in BT and 0.00% in PBT, *p* = 0.018) (Figure 3).

Results above indicated that the effect of prednisone on gut microbiota varied in the presence of bromofuranone. Regardless of the presence or absence of bromofuranone, *Mucispirillum* is the only microbial genus that was reduced by prednisone.

### Alterations in Microbial Genera Associated With Bromofuranone

**FIGURE 4:**
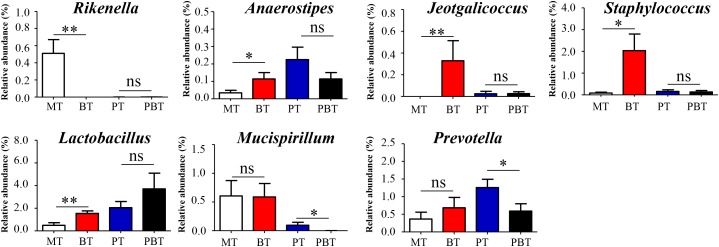
Significantly altered genera are associated with bromofuranone. MT, the model group; PT, the prednisone-treated group; BT, the bromofuranone-treated group; PBT, the combined drugs-treated group. “^∗^”: represents the adjusted *p*-Value < 0.05 between two groups; “^∗∗^”: represents the adjusted *p*-Value < 0.01 between two groups; “ns”: represents the adjusted *p*-Value > 0.05 between two groups.

As an inhibitor of the AI-2/LuxS quorum sensing system, bromofuranone could significantly reduce the abundance of the AI-2 signal in MRL/lpr mice (Supplementary Figure S3). Simultaneously, bromofuranone cause a decrease in *Rikenella* (from 0.49 to 0.0%, *p* < 0.001) and the increase in *Anaerostipes* (from 0.03 to 0.12%, *p* = 0.039), *Jeotgalicoccus* (from 0.0 to 0.37%, *p* < 0.001), *Staphylococcus* (from 0.08 to 2.03%, *p* = 0.017), and *Lactobacillus* (from 0.49 to 1.54%, *p* = 0.006) (Figure 4 and Supplementary Figure S4). Furthermore, the effect of bromofuranone on the gut microbiota was altered in presence of prednisone. Only two genera, *Prevotella* (1.26% in PT and 0.59% in PBT, *p* = 0.032) and *Mucispirillum* (0.10% in PT and 0.01% in PBT, *p* = 0.048), were significantly different between the PT and PBT groups (Figure 4 and Supplementary Figure S4).

### Alterations in Microbial Genera Between the Combination and Model Groups

**FIGURE 5:**
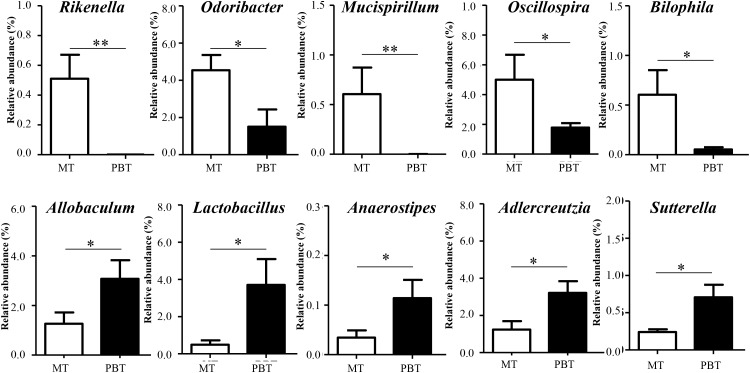
Significantly different genera between MT and PBT groups in MRL/lpr mice. MT, the model group; PBT, the combined drugs-treated group. “^∗^”: represents the adjusted *p*-Value < 0.05 between two groups; “^∗∗^”: represents the adjusted *p*-Value < 0.01 between two groups.

As discussed above in Figure 1, combined drugs showed the best therapeutic effect in MRL/lpr mice, so that it was necessary to reveal the different microbial genera between the PBT and MT groups (Supplementary Figure S5). As shown in Figure 5, 10 genera with relative abundance > 0.10% were significantly different between the two groups. The combined use of the two drugs caused the decrease in *Rikenella* (from 0.51 to 0.0%, *p* = 0.007), *Odoribacter* (from 4.54% to 1.50%, *p* = 0.040), *Mucispirillum* (from 0.53 to 0.0%, *p* < 0.001), *Oscillospira* (from 5.00 to 1.78%, *p* = 0.046), and *Bilophila* (from 0.60 to 0.05%, *p* = 0.036), meanwhile the increase in *Adlercreutzia* (from 1.24 to 3.21%, *p* = 0.026), *Lactobacillus* (from 0.49 to 3.71%, *p* = 0.026), *Anaerostipes* (from 0.03 to 0.11%, *p* = 0.038), *Allobaculum* (from 1.26 to 3.07%, *p* = 0.037), and *Sutterella* (from 0.24 to 0.71%, *p* = 0.001) (Figure 5).

### Correlations of Significantly Altered Genera and Lupus Activity Index

**FIGURE 6:**
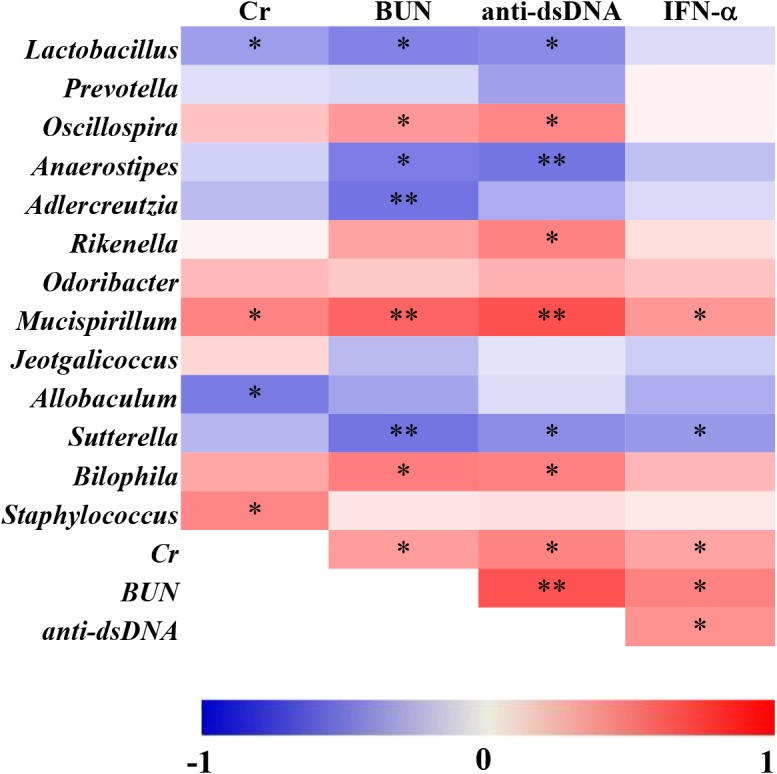
Spearman’s correlation between the relative abundance of altered microbial genera and the indexes of lupus activity in MRL/lpr mice. The data are shown as heatmaps, with the color of each correlation test corresponding to the Spearman rho value: “^∗^” represents *p* < 0.05; “^∗∗^” represents *p* < 0.01.

To further understand the association between microbial genera and lupus activity, Spearman’s rank correlation method was used to explain the correlations of 13 significantly altered genera and lupus activity. As shown in Figure 6, statistically significant positive correlations between microbial genera and lupus activity were identified. Remarkably, all four lupus activity indexes were positively correlated with *Mucispirillum.* There were, as well, statistically significant positive correlations among the following factors: BUN and anti-dsDNA and *Oscillospira*; *Rikenella* and anti-dsDNA; *Staphylococcus* and Cr; and BUN and anti-dsDNA and *Bilophila* (Figure 6). Additionally, this study also identified statistically significant negative correlations between the following: *Lactobacillus* and three lupus activity indexes (Cr, BUN and anti-dsDNA); *Sutterella* and three lupus activity indexes (BUN, anti-dsDNA and IFN-α); BUN and anti-dsDNA and *Anaerostipes*; BUN and *Adlercreutzia*; and Cr and *Allobaculum* (Figure 6).

## Discussion

In recent years, the roles of the gut microbiota in SLE had become increasingly apparent and studies had indicated that the changing of gut microbiota could affect lupus activity (Cuervo et al., 2015; Mu et al., 2017). In this study, the regulation of gut microbiota by bromofuranone did not influence lupus activity but could promote the efficacy of prednisone in mice.

Glucocorticoids suppress immunity and inflammation by regulating the GCs receptor (Cain and Cidlowski, 2017) and gut microbiota (Huang et al., 2015). In MRL/lpr mice, the alterations of gut microbiota were closely related to the efficacy of prednisone. *Mucispirillum*, which was downregulated by prednisone in MRL/lpr mice, could degrade colonic mucin in intestines (Cai et al., 2017). It was reported that dexamethasone could also lead to the depletion of *Mucispirillum* in mice with inflammatory bowel diseases (Huang et al., 2015). Additionally, *Mucispirillum* was positively related to the lupus activity in MRL/lpr mice and the abundance of *Mucispirillum* could be an indicator of collagen induced-arthritis (Ben-Amram et al., 2017). In the prednisone-treated lupus mice, *Anaerostipes* and *Mucispirillum* conversely related with lupus activity. During the progression of lupus in NZB/W F1 model, *Anaerostipes* was also negatively correlated with lupus activity (Luo X. M. et al., 2018a). *Anaerostipes* maintained gut health by producing butyrate, which was the primary source of bacterial energy (Bui et al., 2014). Moreover, the decreased abundance of *Anaerostipes* was associated with multiple sclerosis (Chu et al., 2018). Like the above two genera, prednisone also significantly altered three other genera which were significantly associated with lupus activity. The positive correlations between *Oscillospira*, *Bilophila*, *Rikenella*, and lupus activity was in accordance with a prior study where three genera showed significant increases from the predisease stage to the diseased stage in lupus mice (Luo X.M. et al., 2018). In summary, the prednisone-associated alterations in gut microbiota were associated with lupus activity.

With the addition of bromofuranone, the therapeutic efficacy of prednisone was further promoted in MRL/lpr mice. The prednisone-induced alterations in gut microbiota were also found in the MRL/lpr mice treated with combination therapy. Meanwhile, the combined use of bromofuranone and prednisone induced the other alterations in *Lactobacillus*, *Allobaculum*, *Sutterella*, and *Adlercreutzia*, which were not induced by prednisone alone. It was reported that *Lactobacillus* was beneficial to relieve lupus activity (Mu et al., 2017) by suppressing proinflammatory responses and increasing the number of inducible regulatory T cells (Mohamadzadeh et al., 2011; Khorasani et al., 2018). Moreover, three other specifically altered genera, *Allobaculum*, *Sutterella*, and *Adlercreutzia*, were negatively correlated with lupus activity and were all reported to be capable of immunoregulatory in intestines (Hiippala et al., 2016; Calvo-Barreiro et al., 2018; Scott et al., 2018). The alterations in *Lactobacillus*, *Allobaculum*, *Sutterella*, and *Adlercreutzia* might be associated with the promotability of bromofuranone on the efficacy of prednisone.

Overall, this research demonstrated that the gut microbiota was one of the targets of prednisone for SLE, and inhibiting the AI-2 quorum sensing of gut microbiota by bromofuranone could impact the efficacy of prednisone. This is the first study linking glucocorticoid efficacy with the alterations in gut microbiota during the treatment of SLE. Although further investigation is needed to fully characterize the roles of the significantly altered genera and determine whether QS systems of the gut microbiota is the target of GCs, this study provides an essential step in studying the interaction between gut microbiota and GCs.

## Ethics Statement

All animal handling and experimental procedures were performed in accordance with local ethical committees and the National Institutes of Health Guide for the Care and Use of Laboratory Animals. All efforts were made to minimize animal suffering and to reduce the number of animals used. All procedures performed in this study involving animals were approved by the Ethics Committee of Zhejiang Chinese Medical University.

## Author Contributions

All authors were involved in drafting the article or revising it critically for the important intellectual content, and approved the final version to be submitted for publication. ZH had full access to all of the data in the study and takes responsibility for the integrity of the data and the accuracy of the data analysis. ZH and CW conceived and designed the study. ZH, XK, YZ, and TS analyzed and interpreted the data.

## Conflict of Interest Statement

The authors declare that the research was conducted in the absence of any commercial or financial relationships that could be construed as a potential conflict of interest.

## Footnotes

1 http://huttenhower.sph.harvard.edu/lefse/

## Abbreviations

BT: the bromofuranone-treated group
BUN: blood urea nitrogen
Cr: creatinine
GCs: glucocorticoids
LEfSe: linear discriminant analysis effect size
MT: the model group
PBT: the combined drugs-treated group
PCoA: principal coordinates analysis
PT: the prednisone-treated group
QS: quorum sensing
SLE: systemic lupus erythematosus

## References

[B1] Bassler B. L. Greenberg E. P. Stevens A. M. (1997). Cross-species induction of luminescence in the quorum-sensing bacterium *Vibrio harveyi*. *J. Bacteriol.* 179 4043–4045. 10.1128/jb.179.12.4043-4045.1997 9190823 PMC179216

[B2] Ben-Amram H. Bashi T. Werbner N. Neuman H. Fridkin M. Blank M. (2017). Tuftsin-phosphorylcholine maintains normal gut microbiota in collagen induced Arthritic mice. *Front. Microbiol.* 8:1222. 10.3389/fmicb.2017.01222 28740485 PMC5502260

[B3] Bui T. P. N. de Vos W. M. Plugge C. M. (2014). Anaerostipes rhamnosivorans sp nov., a human intestinal, butyrate-forming bacterium. *Int. J. Syst. Evol. Microbiol.* 64 787–793. 10.1099/ijs.0.055061-0 24215821

[B4] Buren J. Lai Y. C. Lundgren M. Eriksson J. W. Jensen J. (2008). Insulin action and signalling in fat and muscle from dexamethasone-treated rats. *Arch. Biochem. Biophys.* 474 91–101. 10.1016/j.abb.2008.02.034 18328801

[B5] Cai W. Ran Y. Li Y. Wang B. Zhou L. (2017). Intestinal microbiome and permeability in patients with autoimmune hepatitis. *Best Pract. Res. Clin. Gastroenterol.* 31 669–673. 10.1016/j.bpg.2017.09.013 29566910

[B6] Cain D. W. Cidlowski J. A. (2017). Immune regulation by glucocorticoids. *Nat. Rev. Immunol.* 17 233–247. 10.1038/nri.2017.1 28192415 PMC9761406

[B7] Calvo-Barreiro L. Eixarch H. Montalban X. Espejo C. (2018). Combined therapies to treat complex diseases: the role of the gut microbiota in multiple sclerosis. *Autoimmun. Rev.* 17 165–174. 10.1016/j.autrev.2017.11.019 29191793

[B8] Caporaso J. G. Kuczynski J. Stombaugh J. Bittinger K. Bushman F. D. Costello E. K. (2010). QIIME allows analysis of high-throughput community sequencing data. *Nat. Methods* 7 335–336. 10.1038/nmeth.f.303 20383131 PMC3156573

[B9] Chu F. N. Shi M. C. Lang Y. Shen D. H. Jin T. Zhu J. (2018). Gut microbiota in multiple sclerosis and experimental autoimmune encephalomyelitis: current applications and future perspectives. *Med. Inflamm.* 2018:8168717. 10.1155/2018/8168717 29805314 PMC5902007

[B10] Cuervo A. Hevia A. Lopez P. Suarez A. Sanchez B. Margolles A. (2015). Association of polyphenols from oranges and apples with specific intestinal microorganisms in systemic lupus erythematosus patients. *Nutrients* 7 1301–1317. 10.3390/nu7021301 25690419 PMC4344589

[B11] Das I. Png C. W. Oancea I. Hasnain S. Z. Lourie R. Proctor M. (2013). Glucocorticoids alleviate intestinal ER stress by enhancing protein folding and degradation of misfolded proteins. *J. Exp. Med.* 210 1201–1216. 10.1084/jem.20121268 23650437 PMC3674691

[B12] Edgar R. C. (2010). Search and clustering orders of magnitude faster than BLAST. *Bioinformatics* 26 2460–2461. 10.1093/bioinformatics/btq461 20709691

[B13] He Z. X. Shao T. J. Li H. C. Xie Z. J. Wen C. P. (2016). Alterations of the gut microbiome in Chinese patients with systemic lupus erythematosus. *Gut Pathog.* 8:64. 27980687 10.1186/s13099-016-0146-9 PMC5146896

[B14] Hevia A. Milani C. Lopez P. Cuervo A. Arboleya S. Duranti S. (2014). Intestinal dysbiosis associated with systemic lupus erythematosus. *mBio* 5:e1548-14. 10.1128/mBio.01548-14 25271284 PMC4196225

[B15] Hiippala K. Kainulainen V. Kalliomaki M. Arkkila P. Satokari R. (2016). Mucosal prevalence and interactions with the epithelium indicate commensalism of *Sutterella* spp. *Front. Microbiol.* 7:1706. 10.3389/fmicb.2016.01706 27833600 PMC5080374

[B16] Hsiao A. Ahmed A. M. Subramanian S. Griffin N. W. Drewry L. L. Petri W. A. (2014). Members of the human gut microbiota involved in recovery from *Vibrio cholerae* infection. *Nature* 515 423–426. 10.1038/nature13738 25231861 PMC4353411

[B17] Huang E. Y. Inoue T. Leone V. A. Dalal S. Touw K. Wang Y. (2015). Using corticosteroids to reshape the gut microbiome: implications for inflammatory bowel diseases. *Inflamm. Bowel Dis.* 21 963–972. 10.1097/MIB.0000000000000332 25738379 PMC4402247

[B18] Kamareddine L. Wong A. C. N. Vanhove A. S. Hang S. Purdy A. E. Kierek-Pearson K. (2018). Activation of *Vibrio cholerae* quorum sensing promotes survival of an arthropod host. *Nat. Microbiol.* 3 243–252. 10.1038/s41564-017-0065-7 29180725 PMC6260827

[B19] Khorasani S. Mahmoudi M. Kalantari M. R. Lavi Arab F. Esmaeili S. A. Mardani F. (2018). Amelioration of regulatory T cells by *Lactobacillus delbrueckii* and *Lactobacillus rhamnosus* in pristane-induced lupus mice model. *J. Cell Physiol.* 234 9778–9786. 10.1002/jcp.27663 30370554

[B20] Li Z. Y. Fan M. B. Zhang S. L. Qu Y. Zheng S. L. Song J. (2016). Intestinal metrnl released into the gut lumen acts as a local regulator for gut antimicrobial peptides. *Acta Pharmacol. Sin.* 37 1458–1466. 10.1038/aps.2016.70 27546006 PMC5099411

[B21] Luo X. M. Edwards M. R. Mu Q. H. Yu Y. Vieson M. D. Reilly C. M. (2018). Gut microbiota in human systemic lupus erythematosus and a mouse model of lupus. *Appl. Environ. Microbiol.* 84:e2288-17. 10.1128/AEM.02288-17 29196292 PMC5795066

[B22] Luo Y. Y. Zeng B. H. Zeng L. Du X. Y. Li B. Huo R. (2018). Gut microbiota regulates mouse behaviors through glucocorticoid receptor pathway genes in the hippocampus. *Transl. Psychiatry* 8:187. 10.1038/s41398-018-0240-5 30194287 PMC6128920

[B23] Mohamadzadeh M. Pfeiler E. A. Brown J. B. Zadeh M. Gramarossa M. Managlia E. (2011). Regulation of induced colonic inflammation by *Lactobacillus acidophilus* deficient in lipoteichoic acid. *Proc. Natl. Acad. Sci. U.S.A.* 108(Suppl. 1), 4623–4630. 10.1073/pnas.1005066107 21282652 PMC3063598

[B24] Morris D. J. Ridlon J. M. (2017). Glucocorticoids and gut bacteria: “The GALF Hypothesis” in the metagenomic era. *Steroids* 125 1–13. 10.1016/j.steroids.2017.06.002 28624548

[B25] Mu Q. Zhang H. Liao X. Lin K. Liu H. Edwards M. R. (2017). Control of lupus nephritis by changes of gut microbiota. *Microbiome* 5:73. 10.1186/s40168-017-0300-8 28697806 PMC5505136

[B26] Mu Q. H. Zhang H. S. Luo X. M. (2015). SLE: another autoimmune disorder influenced by microbes and diet? *Front. Immunol.* 6:608. 10.3389/fimmu.2015.00608 26648937 PMC4663251

[B27] Pereira C. S. Thompson J. A. Xavier K. B. (2013). AI-2-mediated signalling in bacteria. *FEMS Microbiol. Rev.* 37 156–181. 10.1111/j.1574-6976.2012.00345.x 22712853

[B28] Quast C. Pruesse E. Yilmaz P. Gerken J. Schweer T. Yarza P. (2013). The SILVA ribosomal RNA gene database project: improved data processing and web-based tools. *Nucleic Acids Res.* 41 D590–D596. 10.1093/nar/gks1219 23193283 PMC3531112

[B29] Ridlon J. M. Ikegawa S. Alves J. M. P. Zhou B. Kobayashi A. Iida T. (2013). Clostridium scindens: a human gut microbe with a high potential to convert glucocorticoids into androgens. *J. Lipid Res.* 54 2437–2449. 10.1194/jlr.M038869 23772041 PMC3735941

[B30] Sanz I. Lee F. E. H. (2010). B cells as therapeutic targets in SLE. *Nat. Rev. Rheumatol.* 6 326–337. 10.1038/nrrheum.2010.68 20520647 PMC3934759

[B31] Sayers E. W. Agarwala R. Bolton E. E. Brister J. R. Canese K. Clark K. (2018). Database resources of the national center for biotechnology information. *Nucleic Acids Res.* 44 D7–D19. 10.1093/nar/gky1069 26615191 PMC4702911

[B32] Scott N. A. Andrusaite A. Andersen P. Lawson M. Alcon-Giner C. Leclaire C. (2018). Antibiotics induce sustained dysregulation of intestinal T cell immunity by perturbing macrophage homeostasis. *Sci Transl. Med.* 10:eaao4755. 10.1126/scitranslmed.aao4755 30355800 PMC6548564

[B33] Segata N. Izard J. Waldron L. Gevers D. Miropolsky L. Garrett W. S. (2011). Metagenomic biomarker discovery and explanation. *Genome Biol.* 12 R60. 10.1186/gb-2011-12-6-r60 21702898 PMC3218848

[B34] Sivakumar K. Scarascia G. Zaouri N. Wang T. Kaksonen A. H. Hong P. Y. (2019). Salinity-mediated increment in sulfate reduction, biofilm formation, and quorum sensing: a potential connection between quorum sensing and sulfate reduction? *Front. Microbiol.* 10:188. 10.3389/fmicb.2019.00188 30787924 PMC6373464

[B35] Thompson J. A. Oliveira R. A. Djukovic A. Ubeda C. Xavier K. B. (2015). Manipulation of the quorum sensing signal AI-2 affects the antibiotic-treated gut microbiota. *Cell Rep.* 10 1861–1871. 10.1016/j.celrep.2015.02.049 25801025

[B36] Tsokos G. C. (2011). Mechanisms of disease systemic lupus erythematosus. *N. Engl. J. Med.* 365 2110–2121. 10.1056/Nejmra1100359 22129255

[B37] Waters C. M. Bassler B. L. (2005). Quorum sensing: cell-to-cell communication in bacteria. *Annu. Rev. Cell. Dev. Biol.* 21 319–346. 10.1146/annurev.cellbio.21.012704.131001 16212498

[B38] Wu T. Yang L. N. Jiang J. G. Ni Y. H. Zhu J. W. Zheng X. J. (2018). Chronic glucocorticoid treatment induced circadian clock disorder leads to lipid metabolism and gut microbiota alterations in rats. *Life Sci.* 192 173–182. 10.1016/j.lfs.2017.11.049 29196049

[B39] Zhang H. S. Liao X. F. Sparks J. B. Luo X. M. (2014). Dynamics of gut microbiota in autoimmune lupus. *Appl. Environ. Microbiol.* 80 7551–7560. 10.1128/Aem.02676-14 25261516 PMC4249226

