# Supplementary material for: Alterations of the Gut Microbiota Associated With Promoting Efficacy of Prednisone by Bromofuranone in MRL/lpr Mice
Source: Front Microbiol. 2019 May 1;10:978. doi: 10.3389/fmicb.2019.00978 (PMC6504707; doi:10.3389/fmicb.2019.00978)
Supplement: Supplementary file 1 [file Data_Sheet_1.docx]

**Alterations of the Gut Microbiota Associated with Promoting Efficacy of Prednisone by Bromofuranone in MRL/lpr Mice**

Zhixing He^+^, Xiangyu Kong^+^, Tiejuan Shao, Yun Zhang*, Chengping Wen*

Institute of Basic Research in Clinical Medicine, College of Basic Medical Science, Zhejiang Chinese Medical University, Hangzhou, 310053, China;

*Corresponding author. Address: College of Basic Medical Science, Zhejiang Chinese Medical University, Hangzhou 310053, China.

“+”: these authors contributed equally to this work.

Tel.: 086-571-86613587.

E-mail address: wengcp@yeah.net (Chengping Wen), Yunzhang2018@yeah.net (Yun Zhang)

**Summary of supporting information:**

**A. SUPPORTING FIGURES S1-S5**

**Figure S1** Renal HE staining pathological (x 40) section analysis. A: MT; B: PT; C: BT; D: PBT. MT: the model group; PT: the prednisone-treated group; BT: the bromofuranone-treated group; PBT: the combined drugs-treated group.

**Figure S2** LEfSe identified the differential microbial taxa between control MRL/lpr mice (MT) and prednisone-treated MRL/lpr mice (PT) (A), and between bromofuranone-treated MRL/lpr mice (BT) and combined drug-treated MRL/lpr mice (PBT) (B). Significant differences are shown (LDA score >2).

**Figure S3** Abundance of AI-2 signals in the stool samples of MRL/lpr mice. MT: the model group; PT: the prednisone-treated group; BT: the bromofuranone-treated group; PBT: the combined drugs-treated group.“**”: represents the adjusted *p* value <0.01 between two groups.

**Figure S4** LEfSe identified the differential microbial taxa between control MRL/lpr mice (MT) and bromofuranone-treated MRL/lpr mice (BT) (A), and between bromofuranone-treated MRL/lpr mice (PT) and combined drug-treated MRL/lpr mice (PBT) (B). Significant differences are shown (LDA score >2).

**Figure S5** LEfSe identified the differential microbial taxa between control MRL/lpr mice (MT) and combined drug-treated MRL/lpr mice (PBT). Significant differences are shown (LDA score >2).

**
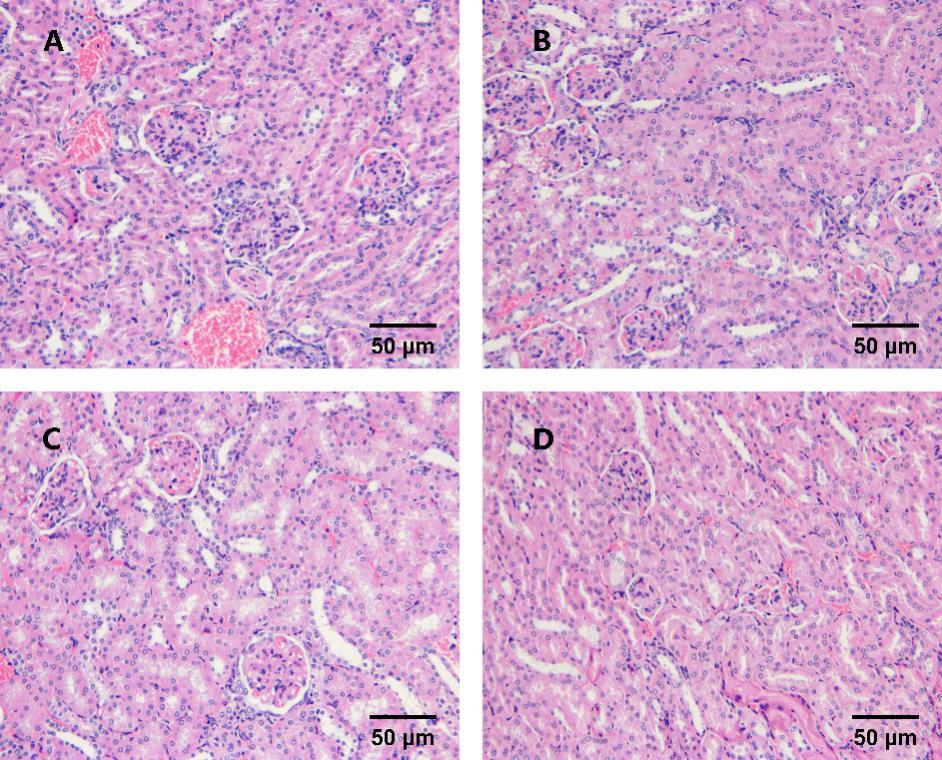
**

**Figure S1** Renal HE staining pathological (x 40) section analysis. A: model group; B: prednisone group; C: bromofuranone group; D: combination group.


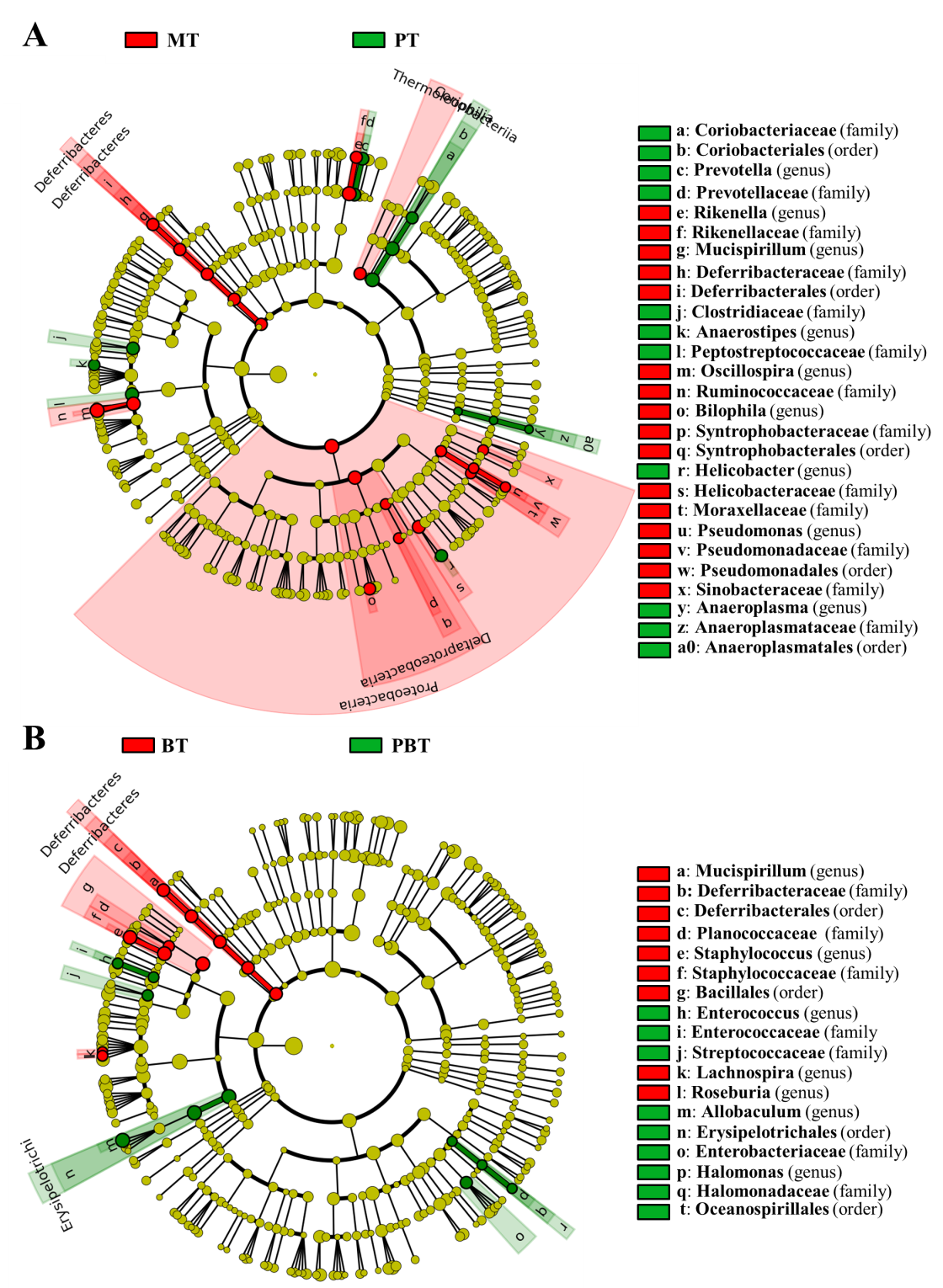


**Figure S2** LEfSe identified the differential microbial taxa between control MRL/lpr mice (MT) and prednisone-treated MRL/lpr mice (PT) (A), and between bromofuranone-treated MRL/lpr mice (BT) and combined drug-treated MRL/lpr mice (PBT) (B). Significant differences are shown (LDA score >2).


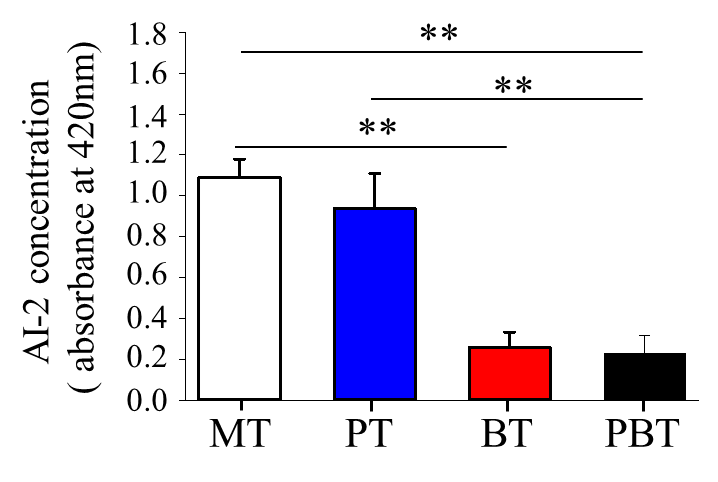


**Figure S3** Abundance of AI-2 signals in the stool samples of MRL/lpr mice. MT: the model group; PT: the prednisone-treated group; BT: the bromofuranone-treated group; PBT: the combined drugs-treated group.“**”: represents the adjusted *p* value <0.01 between two groups.


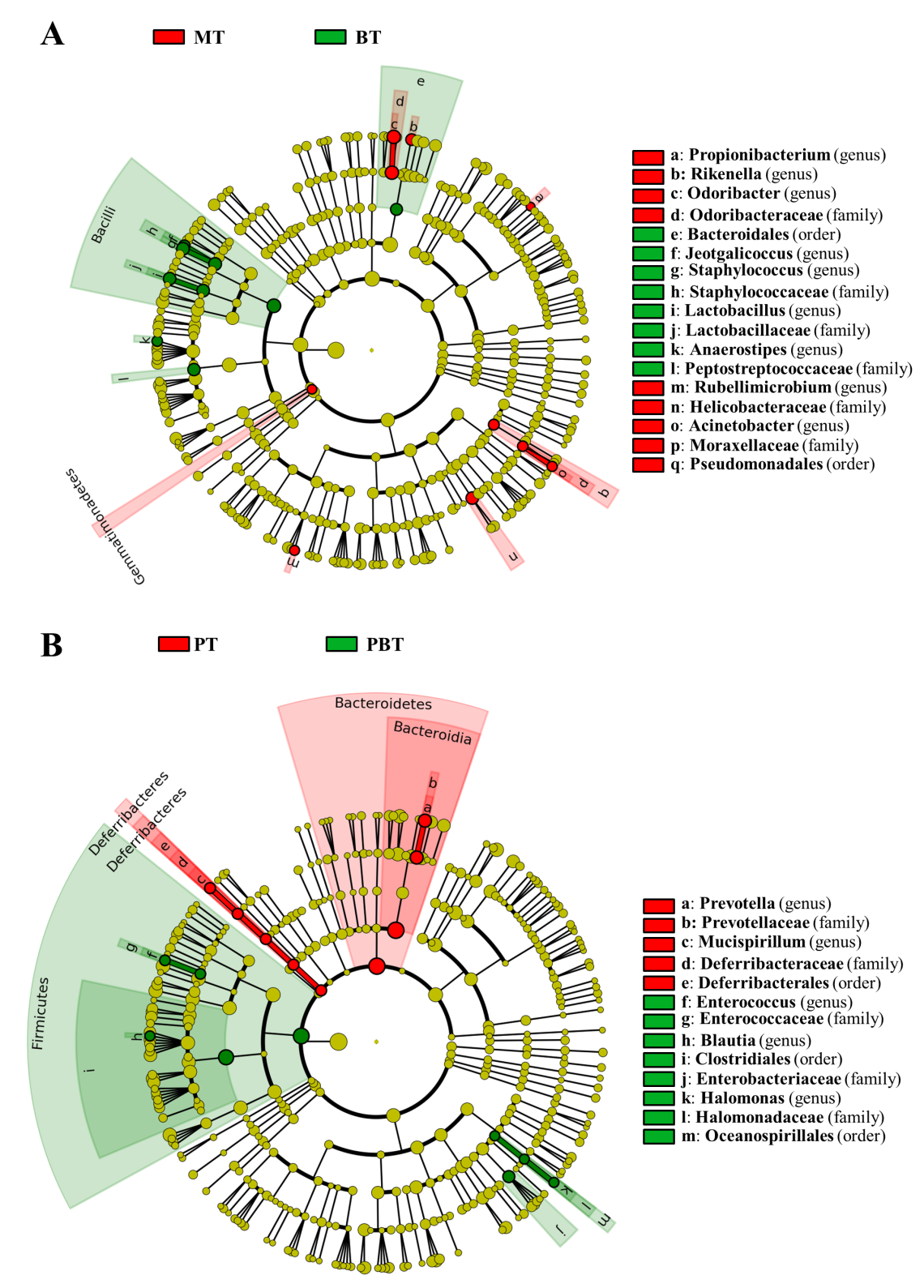


**Figure S4** LEfSe identified the differential microbial taxa between control MRL/lpr mice (MT) and bromofuranone-treated MRL/lpr mice (BT) (A), and between bromofuranone-treated MRL/lpr mice (PT) and combined drug-treated MRL/lpr mice (PBT) (B). Significant differences are shown (LDA score >2).


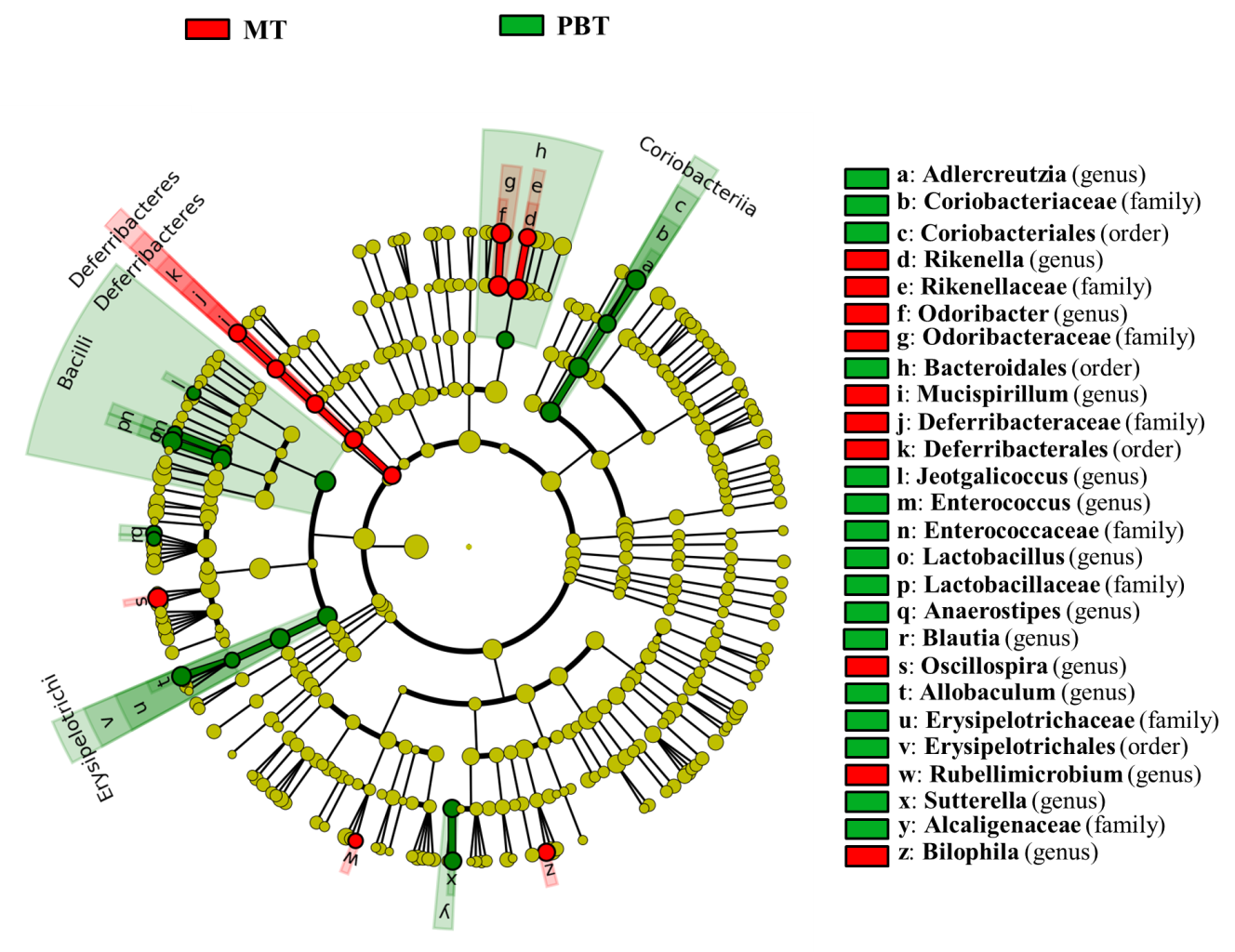


**Figure S5** LEfSe identified the differential microbial taxa between control MRL/lpr mice (MT) and combined drug-treated MRL/lpr mice (PBT). Significant differences are shown (LDA score >2).
